# Sandwich analytics: A dataset comprising one year's weekly sales data correlated with crime, demographics, and weather

**DOI:** 10.1016/j.dib.2019.104252

**Published:** 2019-07-16

**Authors:** Trent J. Spaulding, Edgar E. Hassler, Charles H.L. Edwards, Joseph A. Cazier

**Affiliations:** a Center for Analytics Research and Education, Appalachian State University, USA; b Department of Nutrition and Healthcare Management, Appalachian State University, USA; c Department of Computer Information Systems, Appalachian State University, USA

**Keywords:** Analytics, Marketing, Sales, Food, Forecasting, Sociographic research

## Abstract

Data collected from a quick-serve sandwich chain over one year provide an opportunity to study market, sociographic, meteorologic, and other factors impacting sales and sales forecasting. The weekly sales table contains over 79,000 rows which each represent summary statistics for the sales of an individual menu item in one store during one week of the year. The data were collected from the point-of-sale system of 10 stores. Secondary data regarding weather patterns, population, location, competition, and crime statistics were gathered and integrated with the original data set.

**Table:** Specifications table

Subject area	*Business and Economics*
More specific subject area	*How sales in restaurants are affected by store characteristics, crime, population demographics and weather.*
Type of data	*Six tables: Sales data, store attribute data, county crime data, county demographic data, county employment data, and weather data*
How data was acquired	*Sales and store attribute data provided by the business, secondary data collected from US Census, NOAA Climatic Data Center, Local State managed data portal, Bureau of Labor Statistics, and others.*
Data format	*Anonymized and aggregated raw data*
Experimental factors	*The data represent a natural experiment for the investigation of factors included in the database allowing for a correlative analysis of sales of individual items, location, store features, weather, crime statistics and local demographics.*
Experimental features	*Time, competition, location, weather, local demographics, regional crime statistics and store features.*
Data source location	*Point of sale system of 10 quick-serve stores in two states in the United States*
Data accessibility	https://doi.org/10.17632/6htjnfs78b.1[8]
Related research article	*R. Thomadsen, Product positioning and competition: The role of location in the fast food industry. Marketing Sci. 26(6) (*2007*) 792–804.*

**Table:** 

**Value of the data** • These data provide an opportunity to investigate the effects of weather events on quick-serve sales across stores with different configurations. • Secondary data enables analysis of the effects of county specific variables such as population demographics and crime. These variables can directly and indirectly address socioeconomic and other social factors such as those addressed in [1]. • Competitive pressures on quick-serve sales can be analyzed and explored in this data. Nearby quick-serve and other types of restaurants are included in the data, providing the opportunity to investigate the impact on sales of both. Related work on location and competition has been done in [2], [3]. • The depth and breadth of the data provides additional opportunity beyond the market, sociographic, and meteorological analyses. The data set could also be used as a teaching case for demonstrating forecasting, sociographic interactions and predictive analytical techniques.

## 1 Data

The data were produced from a point-of-sale (POS) system from a quick-serve sandwich chain over the duration of one year. Data were produced by 10 stores which varied in location and other characteristics (see Table 2). The primary sales data and all secondary data are genuine. Nevertheless names, addresses and other key identifying information have been altered to increase anonymity. The secondary data includes demographic data, unemployment data, crime data, weather data, and statistics on other nearby restaurants. Secondary data was primarily collected at the county level, consequently some stores will share the same secondary data. The data set includes three tables: weekly sales, store attributes, and weather reports. All three tables have been anonymized. The datasets contain data for a one-year period from April 2012 to March 2013.

**Table 2:** Other descriptive variables of the quick-serve sandwich stores.

Num	City	County	State	Weather_Station	Location	Drive_Through	Near_School	Competition_Fastfood	Competition_Otherfood
9	Littletown	Appleton	North	Appleton Airport	Strip Mall	No	No	7	18
16	Lake City	Lake	North	Lake City Airport	Strip Mall	Yes	Yes	8	3
2	Power City	Power	North	Rail City Airport	Free Standing	Yes	Yes	10	25
7	Power City	Power	North	Rail City Airport	Strip Mall	No	No	5	6
5	West Power City	Power	North	Rail City Airport	Strip Mall	No	No	1	9
14	Rail City	Rail	North	Appleton Airport	Big Box Retailer	No	No	8	22
11	River City	River	North	River City Airport	Strip Mall	No	Yes	8	30
24	University Town	River	North	River City Airport	Big Box Retailer	No	No	2	1
19	North Town	Farm	South	North Town Airport	Strip Mall	No	Yes	2	20
23	North Town	Farm	South	North Town Airport	Big Box Retailer	No	No	6	18

### 1.1 Weekly sales

The weekly sales data was collected from the chain's POS system, and contains raw data for each item sold in each store for each week, from April 2012 through March 2013 (see Table 1). Profit calculations are included in the dataset, but the costs do not include labor and other variable costs; therefore, profit is gross profit not necessarily an indication of total net profit. However, the owner gave estimates that can be used for labor and approximate rent/lease values for each store, which are included in store attributes table. These could be used as a supplement to estimate net profit and illustrate cost differences in profitability.

**Table 1:** Data descriptions, averages, and standard deviations for the Weekly Sales Table.

Variable	Description	Mean (SD)
Inv_Number	Inventory number of the item sold	
Store_Num	Store number where this item line was sold	
Description	Description of the item sold	
Price	Price of the item sold	4.50 (3.42)
Sold	Quantity sold for this observation, negatives indicate sell backs	21.39 (52.77)
Del	Deliveries entered (denominated in std count units) plus Sum of Transfers In (+) and Transfers Out (−).	0.02 (0.32)
Sales	Sales in dollars for this observation	61.89 (137.41)
Tot_Sls	The percentage of total sales for this week at this store that the item makes up	0.01 (0.01)
Unit_Cost	Cost per unit for the specified inventory item at the specified store	1.09 (1.21)
Cost	Total cost for the observation	19.50 (49.48)
Cost_Percent	Total sales divided by cost	0.23 (0.22)
Margin	Profit margin for this observation	0.01 (0.01)
Profit	Total gross profit for this observation	42.39 (94.47)
Year	Year the item was sold	
Month	Month the item was sold	
Day	Day of the month for the week's observation	

### 1.2 Store attributes

**Fig. 1:**
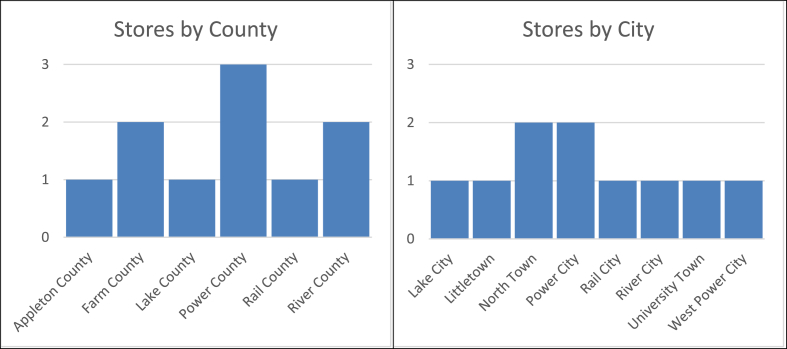
Quick-serve sandwich stores by city and county.

The store attributes table contains descriptive attributes about each store, including location information, information about nearby schools and other restaurants, and demographic and crime information for the county each store is located in. The 10 stores were located in 8 cities across 6 counties (see Fig. 1). The stores include a variety of structures and locations (see Table 2). The chain owner also provided several other data points available in this table. First, stores with more traveler clients are so designated. Second, several stores served higher portions of Hispanic and Native American races. Finally, estimated rent and labor cost is provided for each store.

### 1.3 County data included in the store attributes table

Crime data were collected from the northern state's data portal [4]. Employment data were collected from the Bureau of Labor Statistics Local Area Unemployment [7]. Census data were retrieved from the US Census and use the 2012 Census estimate [5] (see Table 3).

**Table 3:** Employmenst related data and descriptive statistics.

Variable	Description	Mean (SD)
Labor_Force	Total county labor force in 2012	60,407.00 (37,201.80)
Employed	Number of employed persons in 2012	54,592.50 (33,158.93)
Unemployed	Number of unemployed persons in 2012	5,814.50 (4,065.29)
Unemployment_Rate	Percentage of labor force unemployed in 2012	9.28 (1.02)
Crime_Pop	County population from the crime dataset for 2012	105,967.80 (60,598.65)
Total_Crimes	Total number of crimes reported in the county in 2012	8,675.40 (6,207.53)
Total_Crime_Rate	Total crimes per 1000 residents in 2012	75.34 (22.89)
Violent_Crimes	Number of violent crimes reported in the county in 2012	1,551.00 (1,056.97)
Violent_Rate	Violent crimes per 1000 residents in 2012	13.46 (3.71)
Property_Crimes	Property crimes reported in the county in 2012	5,832.80 (4,294.81)
Property_Rate	Property crimes per 1000 residents in 2012	50.98 (17.37)
Society_Crimes	Society crimes reported in the county in 2012	944.00 (685.33)
Society_Rate	Society crimes per 1000 residents in 2012	8.00 (2.04)
Other_Crimes	Other crimes reported in the county in 2012	347.60 (262.24)
Other_Rate	Other crimes per 1000 residents in 2012	2.91 (1.10)
Total_Census_Pop	County population from the 2012 Census estimate	123,861.17 (74,067.21)
Non-Hispanic_White	Number of White residents from the Census estimate in 2012	72,322.17 (41,309.94)
Non-Hispanic_Black	Number of Black residents from the Census estimate in 2012	1,373.83 (0,679.13)
Non-Hispanic_Native_American	Number of Native American residents from the Census estimate in 2012	2,437.17 (3,396.23)
Non-Hispanic_Asian	Number of Asian residents from the Census estimate in 2012	1,942.83 (1,705.55)
Non-Hispanic_Pacific_Islander	Number of Pacific Islander residents from the Census estimate in 2012	144.17 (54.70)
Non-Hispanic_Two_or_more	Number of residents from two or more ethnicities from the Census estimate in 2012	2,256.50 (1,420.96)
Hispanic_White	Number of White residents from the Census estimate in 2012	39,978.83 (33,211.06)
Hispanic_Black	Number of Black residents from the Census estimate in 2012	630.00 (527.69)
Hispanic_Native_American	Number of Native American residents from the Census estimate in 2012	1,436.67 (1,758.56)
Hispanic_Asian	Number of Asian residents from the Census estimate in 2012	289.67 (293.56)
Hispanic_Pacific_Islander	Number of Pacific Islander residents from the Census estimate in 2012	123.50 (113.63)
Hispanic_Two_or_more	Number of residents from two or more ethnicities from the Census estimate in 2012	925.83 (902.81)

Note: N = 6 Counties. Crime variables are missing for one county. County variables are found in the store table with the prefix “county_”.

### 1.4 Weather data

The weather data were collected from NOAA's Climatic Data Center [6]. Eight variables were retained, described in the data description below. Observations exist for each day for five weather stations which are linked back to the sales data by the store attributes table. The weather data consist primarily of wind speed, precipitation, and temperature observations. Descriptive statistics, broken down by weather station, as well as a data dictionary can be found in Table 4. Averages across different weather stations can be found in Table 5.

**Table 4:** Weather related data and descriptive statistics.

Variable	Description	Mean (SD)
Station	Anonymized weather station name	
Date	Date of observation	
Avg_Wind	Average wind speed in miles per hour for week	6.17(2.41)
Precip	Precipitation in inches for week	0.03(0.04)
Snow	Snowfall in inches for week	0.02(0.10)
Snow_Depth	Snow depth in inches for week	0.13(0.74)
Max_Temp	Maximum observed temperature in degrees Fahrenheit for week	64.85(18.81)
Min_Temp	Minimum observed temperature in degrees Fahrenheit for week	40.40(11.43)
Week	Week of the year	
Days_Precipitated	Days in the week where precipitation fell	1.62(1.65)
Days_Snowed	Days in the week which snow fell	0.06(0.37)
Days_With_Snow_Accumulation	Days in the week with snow accumulation	0.08(0.60)
Days_With_Strong_Wind	Days in the week with wind speed above 25mph (25mph is what the NWS considers to be a strong breeze)	0.01(0.11)
Cold_Days	Days in the week where the temperature was below 45° Fahrenheit	1.04(2.02)
Bad_Weather_Days	Days in the week in which it precipitated, snowed, was windy, or below 45°	2.34(2.26)
Bad_Weather_Week	Whether the week had 5 or more bad weather days (Yes/No)

**Table 5:** Mean weather statistics by weather station.

Weather Station	Avg_Wind	Precip	Snow_Depth	Max_Temp	Min_Temp
Appleton Airport	5.02	0.02	0.27	65.66	38.46
Lake City Airport	6.37	0.02	0.00	63.57	40.09
North Town Airport	6.48	0.03	.	65.99	39.84
Rail City Airport	6.05	0.02	0.00	67.00	40.04
River City Airport	7.38	0.05	0.00	64.73	44.58

## 2 Experimental design, materials, and methods

The sales data were captured directly from the subject company's POS reporting system and are produced and shared with permission of the original data stewards with obfuscation of the original stores.

The secondary data were collected from a number of sources. Weather reports were pulled from NOAA's Climatic Data Center for the nearest airport to each store. The store attributes table includes both primary and secondary data: the variables describing the location of the store and proximity to schools were provided by the firm. The variables describing nearby restaurants were manually collected with google maps. Variables describing competition were collected in 2018; therefore, there is a time lag between most of the data and the data related to competitive pressure. We recognize this weakness in the data, but note that generally this competitive pressure does not change quickly.

Crime statistics were pulled from the northern state's data portal, and aggregated into violent, non-violent, property, society, and other crimes. Violent crimes include murder, manslaughter, forcible sex, assault, and kidnapping/abduction. Property crimes were aggregated in the original data, and include arson, bribery, burglary, counterfeiting/forgery, destruction of property, extortion/blackmail, robbery, and theft. Society crimes were also provided in the original data, and include drug violations, pornography, prostitution, weapon violation, and animal cruelty. The remaining reported crimes were aggregated into other crimes and include non-forcible sex and violation of no contact order.

Demographic statistics were retrieved from the US census bureau for the 2012 census estimate on the county level in both states. Unemployment statistics were collected from the Bureau of Labor Statistics’ Local Area Unemployment estimates for 2012.

The sales data were anonymized by renaming menu items and renumbering the stores. Locations were anonymized by changing the names of cities, counties, and states. None of the other data was changed so that any conclusions drawn from the data can still be valid. Keys to de-anonymize the data are held by the authors.

## References

[1] Newman C.L., Howlett E., Burton S. (2014). Implications of fast food restaurant concentration for preschool-aged childhood obesity. J. Business Res..

[2] Thomadsen R. (2007). Product positioning and competition: the role of location in the fast food industry. Mark. Sci..

[3] Shen Q., Xiao P. (2014). McDonald's and KFC in China, Competitors or companions. Mark. Sci..

[4] Local State Statistical Analysis Center. Not Listed to Protect Anonymity of the Data, However This Information Can Be Made Available to Researchers by Contacting the Authors.

[5] (2018). US Census Demographic Data by County.

[6] (2018). NOAA Climatic Data Center Climate Data Online.

[7] (2018). Bureau of Labor Statistics Local Area Unemployment.

[8] Spaulding T.J., Hassler E.E., Edwards C., Cazier J.A. (2019). Sandwich Analytics: A Dataset Comprising One Year's Weekly Sales Data Correlated with Crime, Demographics, and Weather, Mendeley Data, v1.

